# Examining the Effects of Land Use on Carbon Emissions: Evidence from Pearl River Delta

**DOI:** 10.3390/ijerph18073623

**Published:** 2021-03-31

**Authors:** Yabo Zhao, Shifa Ma, Jianhong Fan, Yunnan Cai

**Affiliations:** School of Architecture and Urban Planning, Guangdong University of Technology, Guangzhou 510090, China; zhaoyb@gdut.edu.cn (Y.Z.); whuma@163.com (S.M.)

**Keywords:** land-use-related carbon emissions, influencing factors, LMDI, urban agglomeration, Pearl River Delta

## Abstract

Land-use change accounts for a large proportion of the carbon emissions produced each year, especially in highly developed urban agglomerations. In this study, we combined remote sensing data and socioeconomic data to estimate land-use-related carbon emissions, and applied the logarithmic mean Divisia index (LMDI) method to analyze its influencing factors, in the Pearl River Delta (PRD) of China in 1990–2015. The main conclusions are as follows: (1) The total amount of land-use-related carbon emissions increased from 684.84 × 10^4^ t C in 1990 to 11,444.98 × 10^4^ t C in 2015, resulting in a net increase of 10,760.14 × 10^4^ t (16.71 times). (2) Land-use-related carbon emissions presented a “higher in the middle and lower on both sides” spatial distribution. Guangzhou had the highest levels of carbon emissions, and Zhaoqing had the lowest; Shenzhen experienced the greatest net increase, and Jiangmen experienced the least. (3) The land-use-related carbon emissions intensity increased from 4795.76 × 10^4^ Yuan/t C to 12,143.05 × 10^4^ Yuan/t C in 1990–2015, with the greatest increase seen in Huizhou and the lowest in Zhongshan. Differences were also found in the spatial distribution, with higher intensities located in the south, lower intensities in the east and west, and medium intensities in the central region. (4) Land-use change, energy structure, energy efficiency, economic development, and population all contributed to increases in land-use-related carbon emissions. Land-use change, economic development and population made positive contributions, while energy efficiency and energy structure made negative contributions. At last, we put forward several suggestions for promoting low-carbon development, including development of a low-carbon and circular economy, rationally planning land-use structure, promoting reasonable population growth, improving energy efficiency and the energy consumption structure, and advocating low-carbon lifestyles. Our findings are useful in the tasks related to assessing carbon emissions from the perspective of land-use change and analyzing the associated influencing factors, as well as providing a reference for realizing low-carbon and sustainable development in the PRD.

## 1. Introduction

In the last century, cities have witnessed a transformation whereby they have gradually become the primary location for human settlement on the planet—this is especially true for urban agglomerations, where populations, economic and social activities, economic development, and global–local interactions are all concentrated [1]. Currently, 55% of the world’s population lives in cities, which—although they occupy less than 3% of the surface of the Earth—account for 75% of the total carbon emissions [2]. Global warming, which is caused by the impacts of increasing carbon emissions, has become one of the most serious environmental problems facing humanity today [3]. A recent Intergovernmental Panel on Climate Change (IPCC) report shows that more than 90% of the current global warming is due to the warming effect of greenhouse gases emitted by human activities [4]. As the most concentrated sites for human activities, urban agglomerations have become the main source of carbon emissions [5,6]. Land-use change is considered to be the second largest factor in increasing the global carbon content, after fossil fuel combustion [7]. In addition, human activities such as construction, economic development, industrial activities, urban expansion, and energy consumption are all closely related to land use [8]. Given this, low-carbon land use forms the basis for developing a low-carbon economy. Studying the carbon emissions related to land use (here termed “land-use-related carbon emissions”), as well as the influencing factors behind such emissions, is of great practical significance for realizing low-carbon land use, reducing carbon emissions, and developing a low-carbon economy.

“Carbon emissions” are a general term for the greenhouse gas emissions represented by carbon dioxide. Carbon emissions are one of the main reasons for global climate change [9]. The pressure of public opinion in international climate negotiations and the resource and environmental constraints now being seen in domestic energy conservation and emission reduction efforts have made carbon emissions a widespread and sustained issue of concern amongst policy makers, industry and manufacturing representatives, and researchers in China. The focus of current research on carbon emissions includes: carbon emissions estimation and accounting [10], the effects of carbon emissions on diverse factors and mechanisms of action [11], carbon emission scenario analyses and forecasting [12], carbon emission reduction technology, and policy simulation [13]. Among these endeavors, the analysis of the influencing factors of carbon emissions forms a crucial task, providing vital support to the formulation and implementation of emission reduction policies and scenario simulations.

Land-use change is an important influencing factor in carbon emissions and contributes substantially to global warming [14]. It is estimated that land-use and land-cover change has contributed about one-third of all anthropogenic carbon emissions since the industrial revolution [15]. At a national scale, land-use change led to the production of about 1.45 Pg Cin in China between 1990 and 2010 [16]. Similar results have been found in relation to other countries: in the US, nearly three million hectares of cropland expansion occurred between 2008 and 2012, resulting in a total emission of 38.8 Tg C yr^−1^ [17]; in Brazil, 83% of carbon emissions come from agriculture, land-use change, and deforestation [18]; in South Africa, agricultural land-use is the main source of carbon emissions [19]. Moreover, the transformation of forests to grasslands in New Zealand also holds important implications for carbon emissions, given the different capacities of emission and absorption between the two landscape types [20]. At a regional scale, research into land-use-related carbon emissions can be classified into two categories: studies of cultivated land being transformed into built-up land [21,22,23,24], and studies of forests being transformed into cultivated land [25,26]. Working on the former question, Chuai et al. found that such a transformation contributed more than any other factor to increased carbon emissions in the coastal area of Jiangsu, China [21]. Additionally, this transformation has also been identified as the dominant reason for carbon emission increases in Hangzhou, China [22]. It has been estimated that a 1% increase in the proportion of urban areas resulted in a 0.353–0.592% increase CO_2_ emissions in the Yangtze River Delta [23]. Further, Zhang et al. found the relationship between carbon emission intensity and residential land-use to adopt an inverted-U curve [24], a finding which is consistent with the trends shown in the Environmental Kuznets Curve (EKC). For the latter question, we noted that a study by Baumann et al. found that more than 142,000 km^2^ of the forests in South American Chaco have been replaced by croplands or grazing lands, causing an 824 Tg C increase [25]. By reconstructing land cover from historical records, Li et al. estimated the carbon emissions induced by cropland expansion in northeast China between 1683 and 1980 to be 1.06–2.55 Pg C [26]. There is no doubt that research on land-use-related carbon emissions has achieved fruitful results and these results have acted as a valuable reference for this study. There remain some knowledge gaps that can be studied: (1) The resolution of land-use data is generally not high enough, and many studies have focused on low-resolution remote sensing data, which has constrained the accuracy of the research results, especially in relation to large-scale areas [27,28]; (2) The existing studies have mainly focused on single city or country study areas [17,29,30], neglecting cross-city regions such as urban agglomerations. This is significant, as urban agglomerations represent the areas where land use changes drastically, making them key carbon emission areas, and hence, it is very important to study such regions [22]; (3) Some factors in the agricultural production process have been ignored, including the issue of agricultural machinery use [31].

The logarithmic mean Divisia index (LMDI) is one of the most effective and commonly used methods for analyzing the factors affecting carbon emissions. By applying LMDI in a study in Pakistan, Lin and Raza identified shifts in the population, activity effects, and gross domestic product (GDP) to be the essential factors behind increases in carbon emissions, while carbon intensity and energy intensity effects were found to participate in cutting emissions [32]. Ma et al. also identified economic development as the primary factor increasing carbon emissions in China [33]. In addition, scholars have used LMDI to investigate the factors affecting carbon emissions in China [34]; Colombia [35]; the European Union [36]; Latin America [37]. These previous studies have demonstrated that LMDI is an effective method to analyze the influencing factors behind carbon emissions, hence why the method is so widely used. However, they have continually emphasized the impacts of economic development and energy consumption on carbon emissions, while insufficient attention has been paid to land-use factors, which also exert important influences on carbon emissions.

Greater knowledge about the carbon emissions generated by land-use changes in cross-city regions is crucial to understanding the interaction between land use and carbon emissions, and to the task of policy makers in tackling carbon emissions reduction from a regional perspective. Thus, this paper takes the Pearl River Delta (PRD) urban agglomeration in China as an empirical case, using remote sensing data at a resolution of 30 m and comprehensive socioeconomic data, and applying the land-use carbon emissions estimation and LDMI method to evaluate land-use-related carbon emissions and its influencing factors from 1990 to 2015. The present study not only seeks to provide a reference for regional reductions in land-used-related carbon emissions, but also aims to act as a helpful to guide for evaluating the regional effects of land use on carbon emissions, and support in the task of realizing low-carbon and sustainable development. The remainder of this work is organized as follows: In Section 2, we present the materials and methods; in Section 3, we provide the research findings; in Section 4, we present the discussion; in Section 5, we offer our conclusions.

## 2. Materials and Methods

### 2.1. Study Area

**Figure 1:**
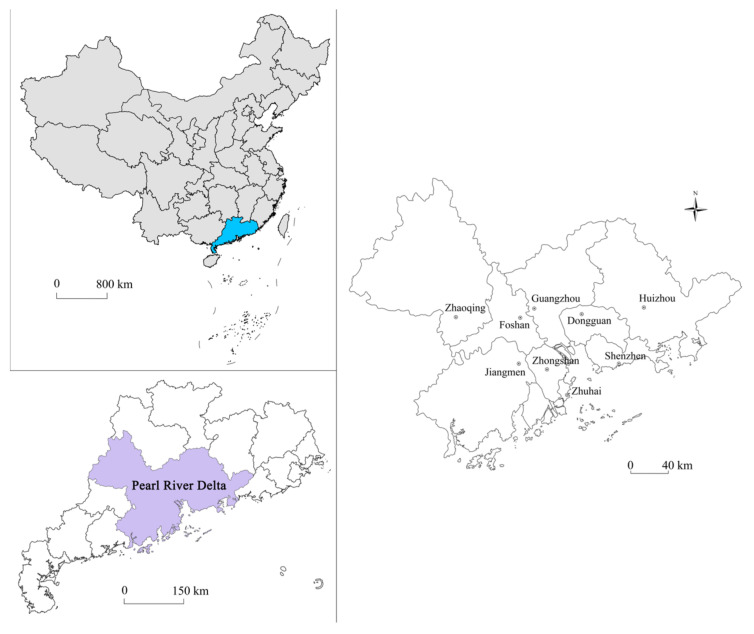
Study area: The Pearl River Delta (PRD).

Our study area comprises of the PRD urban agglomeration (21°17.6′ N–23°55.9′ N, 111°59.7′ E–115°25.3′ E), which falls within the southern subtropics and is located in Guangdong province, in China. With Guangzhou and Shenzhen at its center, the PRD urban agglomeration also includes Zhuhai, Foshan, Jiangmen, Zhaoqing, Huizhou, Dongguan and Zhongshan, with a total of 9 cities in this region (Figure 1).

The PRD urban agglomeration is one of China’s major coastal industrial zones. With the geographical advantages of being in close proximity to neighboring Hong Kong and Macao, the PRD took a step forward through the “Reform and Opening Up” policy of the late 1970s, and gradually developed into an important growth pole in the country. The region covers an area of 54.9 × 103 km^2^, accounting for 0.57% of China; with a population of 58.74 million inhabitants and a GDP of 6.34 trillion Yuan in 2015, contributing 4.27% and 9.20% to the total population and GDP of China, respectively. The population density and per capita GDP of the PRD was 7.47 times and 2.16 times the national average in 2015 (Table 1). However, its rapid industrialization and urbanization has caused dramatic land-use changes, and the on-going development of industry and urban settlements faces challenges and constraints in terms of resources and the environment [38]. The PRD is also under tremendous pressure to achieve carbon emission reductions and hit the sustainable development targets in the “13th Five-Year Plan” (2016–2020).

### 2.2. Data

#### 2.2.1. Land-Use Data

**Figure 2:**
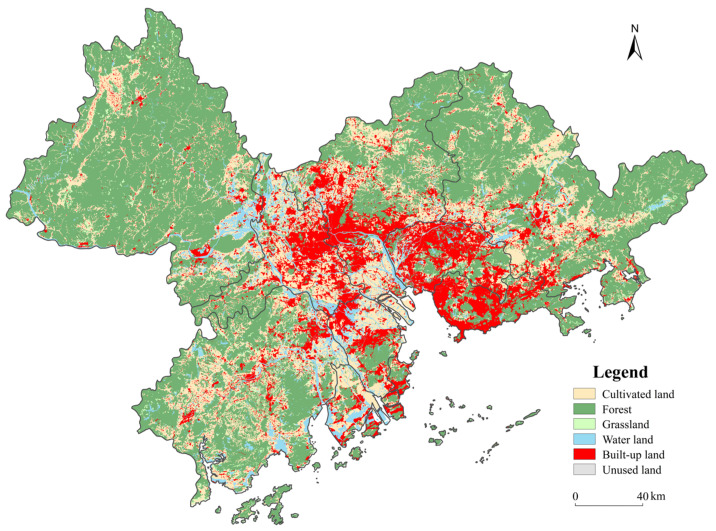
Land use map of PRD in 2015.

Land use is an important factor that affects carbon emissions [21]. To estimate the carbon emissions caused by land-use change, it is essential to first calculate the extent and character of land-use change [39]. We used land-cover data for six periods (1990, 1995, 2000, 2005, 2010, and 2015) with a resolution of 30 m, and included six first-grade land-use types in order to estimate the spatial and temporal dynamics of land use in the PRD. Data were obtained from http://www.resdc.cn (accessed on 15 May 2020). The detailed processes for extracting this dataset can be found in the relevant literature [40]. Furthermore, to test the reliability of the land-use classification results, the Kappa coefficient technique was performed. The results of which were greater than 0.80 for all images, allowing us to conclude that the land-use data used in this study were reliable. Figure 2 shows the spatial distribution of land use in the PRD in 2015.

#### 2.2.2. Socioeconomic Data

Several socioeconomic indicators were selected to assist in calculating the land-use-related carbon emissions and to explore the associated influencing factors. These socioeconomic data were extracted from the China City Statistical Yearbook (1991–2016), the Guangdong Statistical Yearbook (1991–2016), and the statistical yearbooks of all of the cities making up the study area. The coefficient of carbon emissions for different energy sources, the main crops economic coefficient, and the carbon absorption ratio were derived from the IPCC Guidelines for National Greenhouse Gas Emission Inventory (Table 2 and Table 3) [4,9].

### 2.3. Land-Use Carbon Emission/Absorption Estimation

Cultivated land acts as a carbon source in human agricultural production activities, through the use of agricultural fertilizers, agricultural machinery, agricultural films (for instance, the materials used to cover greenhouses, etc.), and irrigation, etc. The following formula is used for calculating the emissions generated in association with cultivated land:

(1) Et=Ef+Em+Ei+Ea

In Formula (1), *E_t_* is the total carbon emissions of cultivated land; *E_f_*, *E_m_*, *E_i_*, *E_a_* is the carbon emissions during the processes of agricultural fertilizer use, agricultural machinery use, agricultural film use, and irrigation. Further, Formula (1) could be decomposed as:

(2) Et=GfA+(AmB+WmC)+AiD+FaE

In Formula (2), *A*, *B*, *C*, *D*, *E* are the conversion coefficients, and their values are 857.54kg/t, 16.47 kg/hm^2^, 0.18 kg/kW, 266.48 kg/hm^2^, 3.84 kg/t, respectively [4,9]. *G_f_*, *A_m_*, *W_m_*, *A_i_*, *F_a_* are the amount of agricultural fertilizer used, crop planting area, total amount of agricultural machinery use, irrigated area, and the amount of agricultural film use, respectively.

While cultivated land is considered to be a carbon sink during the period of crop growth, carbon uptake, mainly from the carbon synthesized by photosynthesis during the growth period, also occurs [41]. The formula for calculating this process is:

(3) $$Ct=\sum_{i}Cdi=\sum_{i}CfiDwi=\sum_{i}CfiYwi/Hi$$

In Formula (3), *C_t_* is the total carbon absorption of cultivated land, *C_di_* is the carbon absorption of crops *i* during the whole growth period, *C_fi_* is the carbon absorption ratio of crops *i*, *D_wi_* is the biological yield of crops *i* (the total accumulation of the substances synthesized by photosynthesis and the substances absorbed by the root system during the life of crops), *Y_wi_* is the economic yield of crops *i* (the part of the biological yield that usually has economic value), and *H_i_* is the economic coefficient of crops *i*. In general, the higher the biological yield, the higher the economic yield [42].

Transportation land is a major carbon source, with the carbon emissions mainly being generated through the use of transportation energy and the process of road construction. The formula for calculating the carbon emissions associated with transportation land is:

(4) $$Ei=\sum_{i}QiCfi$$

In Formula (4), *E_i_* is the total carbon emissions, *Q_i_* is the energy consumption of traffic *i*, and *C_fi_* is the carbon emissions coefficient of traffic energy *i*.

Residential areas, industrial sites, and mining sites are also major carbon sources, with carbon emissions predominantly stemming from industrial energy consumption, human and animal respiration, and household energy consumption. The formula for calculating the carbon emissions associated with residential and industrial mining land is:

(5) $$Ej=\sum_{j}QjCfj+Y\lambda$$

In Formula (5), *E_j_* is the total carbon emissions, *Q_j_* is the energy consumption amount of *j*, *C_fj_* is the carbon emissions coefficient of energy consumption *j*, *Y* is the population, and *λ* is the per capita carbon emissions coefficient.

In addition, land classed as “other cultivated land” is also considered to be a carbon source, with carbon emissions generally being generated through the respiration and excretion of animals. The formula for calculating the emissions generated in association with other cultivated land is:

(6) $$Eo=\sum_{k}Qk\lambda k+S\delta$$

In Formula (6), *E_o_* is the total carbon emissions, *Q_k_* is the livestock and poultry amount of *k*, λ_k_ is the carbon emissions coefficient of livestock and poultry *k*, *S* is the area of other agricultural land and δ is the management coefficient.

Forest, grassland, water bodies, wetlands, and unused land are all generally considered to be carbon sinks. Given that their carbon emission coefficients do not change much over long periods of time, we used existing carbon emission coefficients drawn from previous research to estimate land-use-related carbon emissions. The calculation formula used is:

(7) Ti=Sifi

In Formula (7), *T_i_*, *S_i_* and *f_i_* represent the total carbon emissions, area and carbon emission coefficient of land use type *i*, respectively. The carbon emission coefficient of forest is derived from the research results of Fang et al. [43], while the carbon emission coefficient of water bodies, wetlands, and unused land is derived from Duan et al. [44] and Lai et al. [16]. In addition, the different levels of management and intensity of grassland and the different carbon emission coefficients were taken from the same sources [16]. We considered such coefficients to conform to the category of “improved grassland”.

Carbon intensity refers to carbon emissions per unit *GDP*. Here, this was calculated using the following formula:

(8) $$CI=\frac{GDP}{CE}$$

In Formula (8), *CI* is the carbon emissions intensity, *GDP* is the gross domestic product of a given area, and *CE* is the total carbon emissions.

### 2.4. Land-Use-Related Carbon Emission Modelling Using LMDI

LMDI was initially put forward by Ang [45]. It is able to eliminate the residual term, satisfy the reversible factors, and overcome the shortcomings of the residual term after decomposition or the improper decomposition of the residual term. These advantages make the analysis result more convincing. LMDI is recognized as an accurate method of exponential decomposition and has been widely used in previous studies [46].

Based on the analysis framework of LMDI, and with reference to existing research, the present study selected 4 factors—land-use area, energy consumption, GDP, and population—to analyze the land-use-related carbon emissions that influence factors of the PRD. To this end, we established the following model:

(9) $$\begin{array}{l} E=\frac{E}{A}\times \frac{A}{Q}\times \frac{Q}{GDP}\times \frac{GDP}{P}\times P\\ EI=\frac{E}{A},AI=\frac{A}{Q},QI=\frac{Q}{GDP},GI=\frac{GDP}{P} \end{array}$$

In Formula (9), *E*, *A*, *Q*, and *GDP* represent carbon emissions, land-use area, energy consumption, and *GDP*, respectively; while *EI*, *AI*, *QI*, *GI*, and *P* represent land-use change, energy structure, energy efficiency, the economic level, and population, respectively [47]. For Formula (10), the total carbon emission in the base year is *E*^0^; while it is E^t^ in the year t. In addition, *C_tot_* represents the change during period t. Using addition and decomposition, the expressions of the differential decomposition and the contribution of each decomposition factor are:

(10) $$\Delta Ctot=E^{t}-E^{0}=\Delta EI+\Delta AI+\Delta QI+\Delta GI+\Delta P+\Delta Ersd$$

(11) $$D=\frac{E^{t}}{E^{0}}=DEI\times DAI\times DQI\times DGI\times DP\times Drsd$$

where ∆*EI*, ∆*AI*, ∆*QI*, ∆*GI*, and ∆*P*, refer to the contribution value of each factor (*EI*, *AI*, *QI*, *GI*, and *P*) in terms of land-use-related carbon emissions; *D_EI_*, *D_AI_*, *D_QI_*, *D_GI_*, and *D_P_* are the contribution rates of each factor (*EI*, *AI*, *QI*, *GI*, and *P*), and *D_rsd_* refers to the decomposition residuals. These can be expressed as follows:

(12) $$\begin{array}{l} \Delta EI=\sum \frac{E^{t}-E^{0}}{\ln E^{t}-\ln E^{0}}\ln\frac{EI^{t}}{EI^{0}};\\ \Delta AI=\sum \frac{E^{t}-E^{0}}{\ln E^{t}-\ln E^{0}}\ln\frac{AI^{t}}{AI^{0}};\\ \Delta QI=\sum \frac{E^{t}-E^{0}}{\ln E^{t}-\ln E^{0}}\ln\frac{QI^{t}}{QI^{0}};\\ \Delta GI=\sum \frac{E^{t}-E^{0}}{\ln E^{t}-\ln E^{0}}\ln\frac{GI^{t}}{GI^{0}};\\ \Delta P=\sum \frac{E^{t}-E^{0}}{\ln E^{t}-\ln E^{0}}\ln\frac{P^{t}}{P^{0}};\\ \Delta Ersd=0 \end{array}$$

(13) $$\begin{array}{l} DEI=\exp(W\Delta EI);\;DAI=\exp(W\Delta AI);\\ DQI=\exp(W\Delta QI);\;DGI=\exp(W\Delta GI);\\ DP=\exp(W\Delta P);\;Drsd=1;\\ W=\frac{\ln D}{\Delta Ctot} \end{array}$$

## 3. Results

### 3.1. The Total Amount Changes of Land-Use-Related Carbon Emissions

The total amount of land-use-related carbon emissions increased significantly in the PRD. In 1990, the net carbon emission in this region was only 684.84 × 10^4^ t C, however, this increased sharply to 1889.29 × 10^4^ t C in 1995, and the growth rate reached 175.87%. Since then, the land-use-related carbon emissions have steadily increased, although the growth rate gradually slowed, falling to 130.09% between 1995 and 2000, and then to 65.03% and 36.06% from 2000 to 2005 and 2005 to 2010, respectively. In 2015, the land-use-related carbon emissions reached 11,444.98 × 10^4^ t C, representing a growth of 17.25% from 2010; at this stage, land-use-related carbon emissions were 16.71 times those of 1990 (Table 4). The nine categories of land use that we documented in the PRD can be further divided into two types—“carbon source” and “carbon sink”. The former includes cultivated land, other agricultural land types, residential land, mining and manufacturing land, in addition to transportation land, while the latter includes forest, grassland, water bodies, wetlands, and unused land.

**Figure 3:**
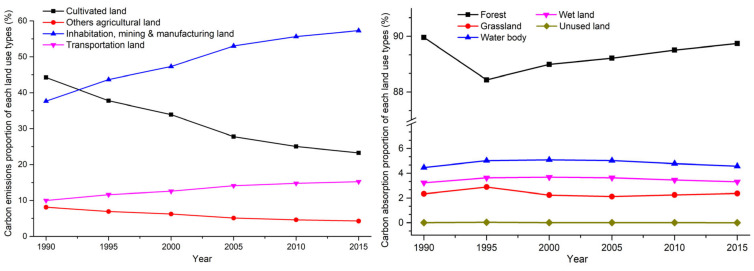
The carbon emissions/absorption of different land-use types in the PRD.

On the one hand, the annual growth in land-use-related carbon emissions and the contribution of the four land-use types that we classified as carbon sources varied significantly (Table 4, Figure 3). Between 1990 and 2015, carbon emissions were found to have increased from 19,918.08 × 10^4^ t C to 29,985.16 × 10^4^ t C—an increase of 10,067.08 × 10^4^ t C—and the growth rate reached 50.54%. Furthermore, among the four carbon source land-use types, whilst the carbon emissions generated in association with cultivated land and other agricultural land decreased gradually, the carbon emissions resulting from residential land, mining and manufacturing land, and transportation land increased rapidly. Cultivated land made the greatest contribution to carbon emissions in 1990, accounting for 44.25% of the total carbon emissions. However, this was exceeded by residential land and mining and manufacturing land in 1995, which together accounted for 43.68%, exceeding the 37.78% contributed by cultivated land. Since 1995, residential land and mining and manufacturing land have become the primary carbon sources, followed by cultivated land, transportation land, and other agricultural land.

On the other hand, carbon absorption in the PRD was found to be relatively stable, with the contribution of the five land-use types also appearing to be stable (Table 4, Figure 3). The carbon absorption remained at around 19,000 × 10^4^ t C throughout the study period, experiencing a minimal reduction from 19,233.24 × 10^4^ t C in 1990 to 18,540.19 × 10^4^ t C in 2015, a variation of just 693.06 × 10^4^ t C and 3.6% over 25 years. In addition, among the five land-use types, forests were shown to absorb the most carbon, accounting for about 89%, while the contributions of the other four categories’ were relatively small, at no more than 6%. In contrast to our carbon emission results, the status of these five land-use types remained unchanged between 1990 to 2015, with forest accounting for the greatest carbon absorption, water bodies representing the second largest, followed by wetland and grassland, while unused land absorbed the least carbon.

### 3.2. Spatiotemporal Evolutions of Land-Use-Related Carbon Emissions

**Figure 4:**
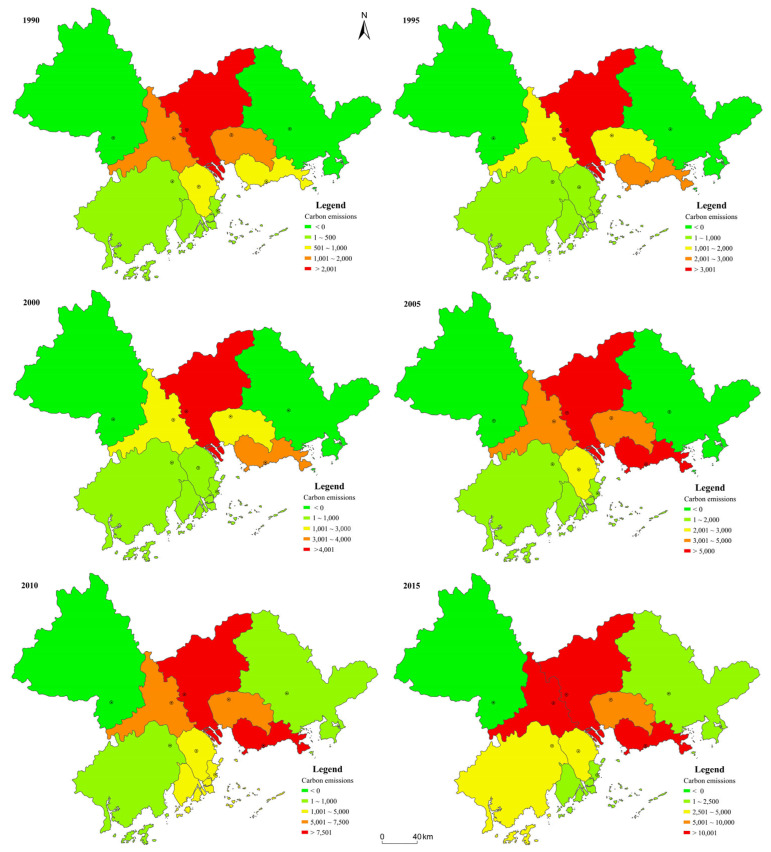
Spatiotemporal changes in land-use-related carbon emissions in the PRD (10^4^ t C).

From a spatial perspective, the spatial distribution of land-use-related carbon emissions in the PRD presents a “higher in the middle and lower on the sides” pattern (Figure 4). Spatially, higher carbon emissions values were found to be mainly located in Guangzhou, Shenzhen, Foshan, and Dongguan, in the middle of the PRD, while lower values tended to be scattered on the eastern and western sides in cities such as Huizhou, Zhaoqing, and Jiangmen.

The spatiotemporal distribution of land-use-related carbon emissions changed significantly in the PRD. Among the nine cities, Guangzhou’s land-use-related carbon emissions remained the highest from 1990 to 2015, while Zhaoqing’s emissions remained the lowest. In 1990, Guangzhou was followed by Foshan in terms of amount of carbon emissions, which was then followed by Dongguan, Shenzhen, Zhongshan, Zhuhai, Jiangmen, Huizhou, and Zhaoqing. However, this sequence changed in 1995, with Shenzhen jumping from fourth to second rank, resulting in Foshan and Dongguan dropping a spot, with the others remaining unchanged. In 2000, Dongguan exceeded Foshan, as the two cities switched places. In 2005, the order of cities was same as in 2000. In 2010, Foshan exceeded Dongguan; they switched back. In 2015, Foshan exceeded Shenzhen, ranking second. Despite these shifts, Shenzhen, Foshan, and Guangzhou witnessed the greatest increases in land-use-related carbon emissions during the study period. Specifically, Shenzhen’s carbon emissions increased from 713.38 × 10^4^ t C in 1990 to 11,037.87 × 10^4^ t C in 2015, resulting in a net increase of 10,324.49 × 10^4^ t C, 14.47 times that of 1990. Foshan’s carbon emissions increased by 10,012.04 × 10^4^ t C, reaching a level that was 7.62 times that of 1990, whilst the emissions level of Guangzhou increased by 9294.37 × 10^4^ t C, with the 2015 rate 2.73 times that of 1990. The cities of Zhaoqing, Zhuhai, and Jiangmen experienced the lowest land-use-related carbon emission increases.

Similarly to the land-use types, the cities of the PRD can be divided into two categories according to their contribution to land-use-related carbon emissions and subsequently classified as either “carbon source cities” or “carbon sink cities”. The carbon source city category—cities release more carbon dioxide than they absorb and exist in a state of net carbon emissions—encompassed the majority of cities in the PRD, including Guangzhou, Shenzhen, Dongguan, Foshan, Zhongshan, Zhuhai, and Jiangmen. Only Zhaoqing and Huizhou were classified as carbon sinks. It is noticeable, however, that the carbon source cities exceeded the carbon sink cities not just in terms of the quantity of emissions, but also the scale and growth rate; this is helpful in understanding why land-use-related carbon emissions in the PRD increased gradually year by year.

### 3.3. Land-Use-Related Carbon Emissions Intensity in the PRD

**Figure 5:**
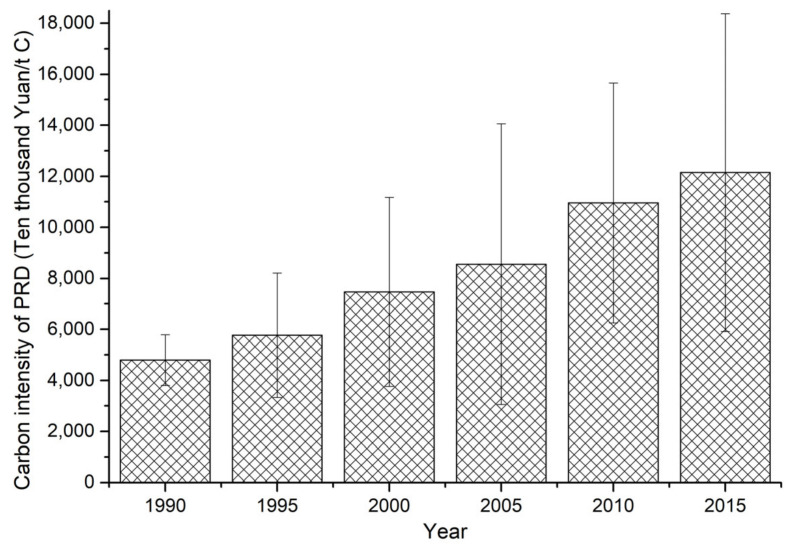
Average land-use-related carbon emissions intensity in the PRD. Note: Error bars show the standard deviation across the region.

The intensity of land-use-related carbon emissions continued to increase from 1990 to 2015 in the PRD (Figure 5). At a regional scale, land-use-related carbon emissions increased from 4795.76 × 10^4^ Yuan/t C in 1990 to 12,143.05 × 10^4^ Yuan/t C in 2015, representing an increase of 7347.29 × 10^4^ Yuan/t C; a 1.53-fold increase over the 25-year period. At the city scale, a positive correlation existed between land-use-related carbon emissions intensity and the economic development level of the nine cities. Huizhou, Shenzhen, and Guangzhou demonstrated the greatest increases in land-use-related carbon emissions intensity, while Zhaoqing, Foshan, and Zhongshan demonstrated the lowest increases.

**Figure 6:**
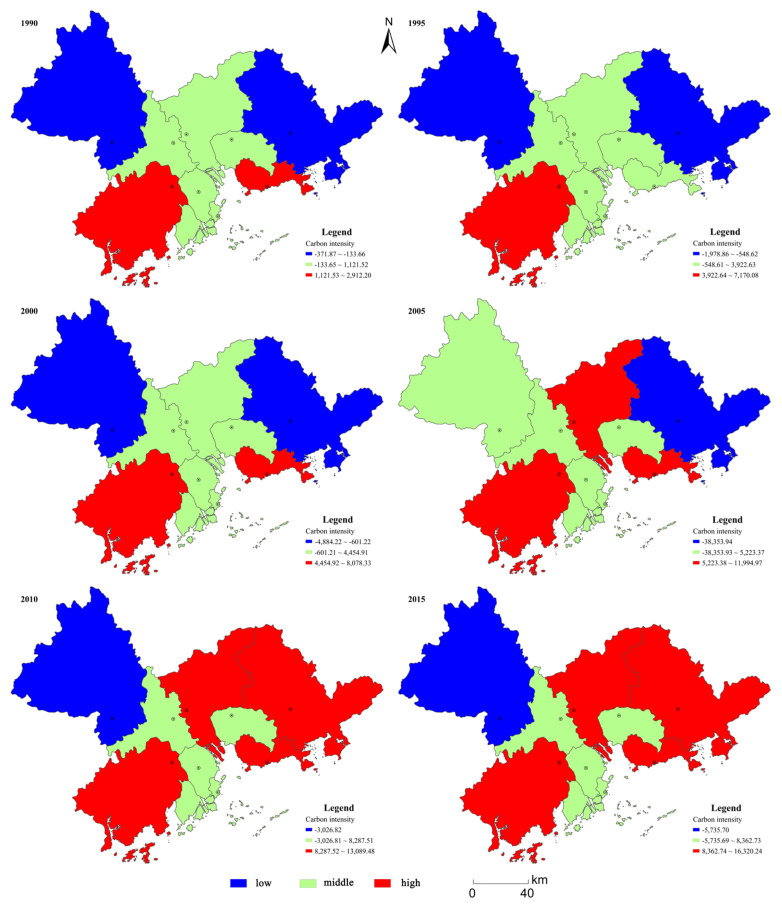
The spatial distribution of dynamic carbon emission intensity in the PRD.

To analyze the spatial distribution and the evolution of land-use-related carbon emissions intensity in the PRD, we mapped the spatial distribution patterns of the selected 6 years (Figure 6). The results demonstrate that significant differences existed. In order to distinguish further differences in the spatial distribution, we broke land-use-related carbon emissions intensity into three grades using the Jenks Classification within ArcGIS. These grades were high, middle, and low.

Land-use-related carbon emission intensity was found to vary significantly in the PRD, exhibiting striking differences not only with respect to amount, but also in terms of spatial distribution. In 1990, quantity-wise, of the nine individual cities, Jiangmen, Shenzhen, and Zhuhai ranked highest, while Zhaoqing, Huizhou and Dongguan ranked the lowest. The spatial distribution pattern was characterized by higher intensities in the south, lower intensities in the east and west, and medium intensities in the central region, surrounded by higher and lower regions. The spatial distribution pattern retained the same order in 1995, showing a distribution pattern made up of higher intensities in the southwest, lower intensities in the east and west, and medium intensities in the central areas. However, a difference was noted, in that the high intensity areas shrunk compared with 1990, and the medium intensity areas expanded. Shenzhen was the reason for this change. In 2000, Jiangmen and Shenzhen were still located the top two, but this was the year that Foshan exceeded Zhuhai and became the third highest, followed by Guangzhou, Zhongshan, and Zhuhai. The lowest cities still maintained the same order as before, but the spatial distribution pattern continued to change and go back to the same situation as in 1990. In 2005, the top two and bottom two were still consistent with the previous years, but the middle part of the ranking had changed, giving the new sequence of Guangzhou > Zhuhai > Foshan > Dongguan > Zhongshan. In terms of the spatial distribution, the areas of high and medium intensity expanded, and Guangzhou entered the high intensity area, Zhaoqing entered the medium intensity area, while the low intensity areas shrunk, with only Huizhou remaining in this grouping. In 2010, we witnessed a new change: Shenzhen registered the highest land-use-related carbon emissions intensity, Guangzhou registered the second highest, followed by Jiangmen, Huizhou, Zhuhai, Foshan, Dongguan, Zhongshan, and Zhaoqing. The most significant change in the spatial distribution was the expansion of the high intensity area to occupy most of the PRD, while the medium intensity areas shrunk. The reason for this phenomenon, was that Huizhou moved from being a low intensity emitter to a high intensity emitter and Zhaoqing moved from being a medium to low intensity emitter. The sequence of cities was relatively stable in 2015, with land-use-related carbon emission intensity registering only minor changes: Shenzhen and Guangzhou remained the two highest intensity emitters, and Zhaoqing the least. The spatial distribution pattern in 2015 was consistent with that of the former year.

### 3.4. Decomposing the Influencing Factors of Land-Use-Related Carbon Emissions

**Figure 7:**
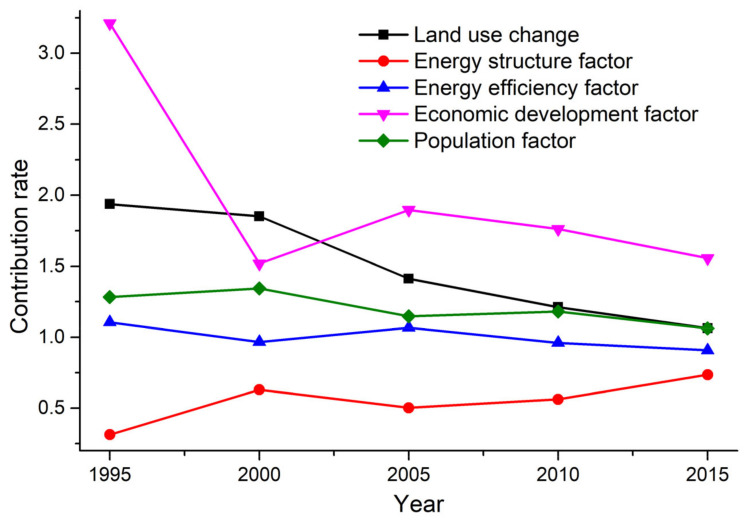
The contribution of decomposed influencing factors on land-use carbon emission in PRD. Note: 1995 denotes the contribution rate of each factor from 1990 to 1995, with other periods following this logic.

By applying the LMDI model, we decomposed the land-use-related carbon emissions of the PRD into five factors: land-use change (∆*EI*), energy structure (∆*AI*), energy efficiency (∆*QI*), economic development (∆*GI*), and population (∆*P*). The contribution of each factor to land-use-related carbon emissions is shown in Table 5 and Figure 7.

Different factors made different contributions to land-use-related carbon emissions in the PRD. Between 1990 and 2015, economic development had the greatest positive impact on the land-use-related carbon emissions increase, resulting in 15,670.63 × 10^4^ t C; this was followed by land-use change and population factors, which resulted in 6806.32 × 10^4^ t C and 3968.73 × 10^4^ t C. In contrast, energy efficiency and energy structure factors were shown to have mitigated land-use-related carbon emissions by eliciting reductions of 982.48 × 10^4^ t C and 14,703.05 × 10^4^ t C. In summary, together, economic development, land-use change and population caused carbon emissions to increase by 26,445.68 × 10^4^ t C, while energy efficiency and energy structure contributed to a 14,703.05 × 10^4^ t C reduction. In combination, the net increase in land-use-related carbon emissions as the result of all five factors was 10,760.15 × 10^4^ t.

We further analyzed the influence exerted by a range of factors on land-use-related carbon emissions in the PRD between 1990 and 2015. Our findings can be described as follows:

(1) Economic development was the primary influencing factor in relation to land-use-related carbon emissions, contributing 15,670.63 × 10^4^ t to carbon emissions increases and accounting for as much as 59.26% of the total. However, we should not ignore the fact that its contribution rate to land-use-related carbon emissions actually decreased from 3.21 in 1990–1995 to 1.56 in 2010–2015. Our results still serve to confirm the positive influence of economic development in enhancing land-use-related carbon emissions.

(2) Land-use change was the second-highest influence in relation to land-use-related carbon emission increases. It contributed 6806.32 × 10^4^ t in carbon emission increases and accounted for 25.74% of the overall effect. The contribution rate of land-use change to land-use-related carbon emissions declined year by year, and the contribution rates for each year of the five year period were 1.94, 1.85, 1.41, 1.21, and 1.06, respectively. Thus, land-use change played a positive, but limited, role in increasing land-use-related carbon emissions.

(3) Population size was found to contribute 3968.73 × 10^4^ t in land-use-related carbon emissions increases, accounting for 15.01% of the overall effect, meaning that it exerted less influence on land-use-related carbon emission increases than economic development and land-use change. Whilst, similarly to the other two factors, the contribution rate also declined, decreasing from 1.28 in 1990–1995 to 1.06 in 2010–2015, population size remains a positive influencing factor in relation to land-use-related carbon emission increases.

(4) In contrast to the three preceding factors, energy structure was found to exert a negative influence in relation to increases in land-use-related carbon emissions, reducing carbon emissions by 14,703.05 × 10^4^ t between 1990 and 2015 and accounting for 93.74% of the total reduction. This finding indicates that energy structure was the most important positive factor contributing to land-use-related carbon emission reductions. This reduction was caused by the increasing use of clean energy and a reduced consumption of raw coal and coke. In addition, the contribution rate of energy structure to land-use-related carbon emissions was always less than one, and showed a growth trend, despite fluctuating slightly in 2000–2005.

(5) Energy efficiency was found to exert a fluctuating influence on land-use-related carbon emissions, but played a negative role overall. During 1990–1995 and 2000–2005, energy efficiency made a positive contribution to land-use-related carbon emission increases, with a contribution value of 117.98 × 10^4^ t and 365.31 × 10^4^ t, respectively. However, it had a negative influence in 1995–2000, 2005–2010, and 2010–2015, when the contribution values were −100.23 × 10^4^ t, −344.71 × 10^4^ t, and −1020.82 × 10^4^ t, respectively. Overall, energy efficiency had a positive influence on land-use-related carbon emission reductions, and the contribution value was 982.48 × 10^4^ t. While its influence fluctuated, the contribution rate of energy efficiency was also proven to be unstable—it was more than one during the period when it generated a positive contribution to land-use-related carbon emission increases. Conversely, in the opposite scenario, it was less than one.

## 4. Discussion

Land-use-related carbon emissions are an important component of carbon emissions and are closely connected with global warming [48]. Despite this, they have seldom been addressed in cross-city regions (for instance, urban agglomeration), even though cross-city regions represent key carbon emission areas. Therefore, it is valuable to study these areas, either directly, or by using high-resolution remote sensing data combined with socioeconomic data. The present paper bridges this gap by documenting an empirical study that used 30 m × 30 m resolution remote sensing data, combined with socioeconomic data, and used the PRD urban agglomeration area in China as the study area. Comparing our results with those of other studies that also used the PRD as a case study area, we found a difference among them. For example, Wang et al. found that the evolution of the interactive coercive relationship between urbanization and carbon emissions conforms to an inverted-U curve, while different aspects of urbanization have different impacts on carbon emissions [7]. Additionally, Xu et al. further pointed out that economic urbanization has the most significant impact on carbon emissions, followed by spatial urbanization, while population urbanization has the lowest impact [49]. Chen et al.’s study revealed that economic growth triggered rapid carbon emission increases in China’s Pearl River Delta (PRD) [50]. Further improvements in some areas still remain, for example, we acknowledge that the method for estimating land-use-related carbon emissions and carbon emission coefficients was based on existing research results [16,43]. Additionally, we focused on the impact of land-use on carbon emissions, neglecting the influence of energy consumption [33]. However, the findings of this study are of importance, forming a reference for low-carbon development in the PRD. Based on the results of this study, the following suggestions for promoting low-carbon development can be made.

(1) The economic development mode must be transformed in order to comprehensively promote a low-carbon and circular economy. The high-energy, high-pollution economic development mode that has characterized the PRD is an important cause of the increase in land-use-related carbon emissions. Thus, the PRD should engage in changing the current economic development model by enhancing the role of science and technology in promoting economic development, optimizing the industrial structure, increasing the proportion of tertiary industry. Additionally, the PRD should adopt “saving resources and protecting the environment” as a development goal, and explore the development of a low-carbon circular economy, with a low-energy, low-pollution, high-tech profile.

(2) The rational planning of land-use structures represents an important method for achieving the goal of land-use-related carbon emission reductions in the PRD. On the one hand, cultivated land needs to be protected and low-carbon agriculture must be vigorously developed. This will involve strictly controlling the occurrence of non-agricultural activities in order to achieve the purpose of land-use-related carbon emission reductions; actively developing new technologies, including the use of new farming techniques; reducing the use of pesticides and fertilizers, making farmland ecosystems more stable. On the other hand, efforts must be made to revitalize the existing stock of land and stimulate the utilization potential of existing land through urban renewal and increasing the utilization efficiency of construction land [51]. The addition of new construction land and the conversion of cultivated land into construction land should be strictly controlled. Additionally, the carbon emission intensity of construction land, especially for industrial land, must be reduced. Moreover, forestland, grassland, and water bodies within the PRD should also be protected.

(3) Reasonable population growth should be promoted. Although the influence of population size on land-use-related carbon emissions is relatively weak, it cannot be ignored. In view of the large population base in the PRD, the degree of attraction of talent to cities, the liberalized two-child policy, as well as the large number of migrant workers in the PRD, it is expected that the population of the PRD will further increase in the future. Attention must therefore be paid to concomitant increases in carbon emissions. Given this, the PRD should strictly implement the government’s new two-child policy, rationally controlling the number of migrants and striving to improve conditions for the population and to promote the reasonable growth of the resident population. Collectively, such measures should reduce the influence of population factors on land-use-related carbon emissions.

(4) Energy efficiency and the energy consumption structure should be improved. The PRD could improve the efficiency of energy consumption by increasing investment in scientific research, technological innovation, and increasing the use of clean energy sources, such as solar energy, wind energy, nuclear energy, etc. in order to improve the energy structure.

(5) Low-carbon planning should be undertaken and low-carbon lifestyles should be advocated in the PRD. The government should ensure that continuous improvements are made to public transportation, subway networks and intercity rail lines, to make travel in and between cities more convenient. Low-carbon buildings, green buildings, and energy-efficient buildings should be comprehensively encouraged, and investment should be transferred to low-carbon, energy-efficient buildings [52]. The transformation from high-consumption and high-energy lifestyles, to low-carbon and environmentally friendly lifestyle should be actively promoted.

## 5. Conclusions

The PRD urban agglomeration in China has witnessed rapid economic and social development since the reform and opening up; however, behind its success lies a number of serious eco-environmental issues, of which, land-use-related carbon emission is one. Similarly to other urban agglomerations around the world, the PRD faces the challenge of realizing low-carbon, sustainable development. Thus, a better understanding of the effects of land use on carbon emissions, as well as the influencing factors at work in the production of land-use-related carbon emissions, is of profound theoretical and practical significance. This study has investigated these two issues through the comprehensive use of remote sensing and socioeconomic data for the 1990–2015 period, via the development of a method for estimating land-use-related carbon emissions and the LMDI model. The detailed results of this research reveal a series of findings, which can be described as follows.

Firstly, the total amount of land-use-related carbon emissions increased significantly. In 2015, land-use-related carbon emissions reached 11,444.98 × 10^4^ t, which was 16.71 times higher than in 1990. Furthermore, we divided nine land-use types into “carbon source” and “carbon sink” categories. The former included cultivated land, other agricultural land types, residential land, mining and manufacturing land, and transportation land, with our analyses showing that their contribution to land-use-related carbon emissions increased year by year. Meanwhile, the latter category included forest, grassland, water bodies, wetlands, and unused land, which achieved relatively stable levels of carbon absorption. Overall, land-use-related carbon emissions remained higher than carbon absorption throughout the study period.

Secondly, land-use-related carbon emissions underwent spatiotemporal changes. Spatially, the distribution was found to present a “higher in the middle and lower on the sides” pattern. Quantitatively, among the nine cities in the PRD, Guangzhou emitted the most carbon, while Zhaoqing emitted the least. Additionally, Shenzhen experienced the greatest net increase and Jiangmen recorded the lowest.

Thirdly, land-use-related carbon emission intensity continually increased, increasing from 4795.76 × 10^4^ Yuan/t C in 1990 to 12,143.05 × 10^4^ Yuan/t C in 2015, representing an increase of 1.53 times. The cities of Huizhou, Shenzhen, and Guangzhou demonstrated the highest increases, while Zhaoqing, Foshan, and Zhongshan experienced the lowest increases. The spatial distribution of land-use-related carbon emission intensity presented a pattern that was characterized by higher intensities in the south, lower intensities in the east and west, and medium intensities in the central regions, surrounded by higher and lower regions.

Fourthly, land-use change, energy structure, energy efficiency, economic development, and population size were identified as the main factors influencing the land-use-related carbon emissions of the PRD. Economic development, land-use change, and population size made a positive contribution to land-use-related carbon emission increases, with economic development exerting the greatest influence. Meanwhile, energy efficiency and energy structure exerted a negative influence, with the contribution of energy structure to land-use-related carbon emission reductions far exceeding that of energy efficiency.

Last but not the least, we attempt to put forward some improvement directions for future research. (1) It is necessary to consider more elements and improve the calculation method of carbon emissions in order to promote the accuracy of the method. (2) We recommend improving the resolution of the land use data, e.g., using a resolution of 10 m, 1 m, or higher [53], in order to increase the accuracy of the data.

## Figures and Tables

**Table 1:** Statistics for the PRD urban agglomeration (2015).

Category	PRD	China	% of China
Area (km^2^)	54,900	9,600,000	0.57
Population (10,000)	5874.28	137,462	4.27
Population density (person/km^2^)	1070.00	143.23	747.05
GDP (billion Yuan)	6338.19	68,905.21	9.20
GDP per capita (Yuan)	107,897.2	49,992	215.83
Urbanization (%)	84.23	56.1	150.14

Source: http://tongji.cnki.net/kns55/Navi/Yearbook.aspx?id=N2017100312&floor=1 (accessed on 6 July 2020).

**Table 2:** Coefficient of carbon emissions for different energy sources.

Energy	Coefficient of Carbon Emissions (t/tce)	Energy	Coefficient of Carbon Emissions (t/tce)	Energy	Coefficient of Carbon Emissions (t/tce)
Raw coal	0.7559	Gasoline	0.5538	Coke oven kerosene	0.3548
Coke	0.8550	kerosene	0.5714	Blast furnace kerosene	0.3548
Washed coal	0.7559	Diesel	0.5921	Other coking products	0.6449
Other coal washing	0.2155	Fuel oil	0.6185	Other gas	0.3548
Briquette	0.4691	Liquefied petroleum gas	0.5042	Other fuel	0.7559
Refinery dry gas	0.4602	Other petroleum products	0.5857	Electric power	2.5255
Crude	0.5857	Natural gas	0.4483	Heat	0.26

**Table 3:** Economic coefficient and carbon absorption ratio for main crops.

Name	Rice	Wheat	Maize	Sorghum	Millet	Potato	Soy	Cotton	Rapeseed	Sunflower	Peanut	Sugar Cane	Tobacco	Others
H	0.45	0.4	0.4	0.35	0.4	0.7	0.34	0.1	0.25	0.3	0.43	0.5	0.55	0.4
C	0.4144	0.4835	0.4709	0.45	0.45	0.4226	0.45	0.45	0.45	0.45	0.45	0.45	0.45	0.45

Note: H = the main crops economic coefficient; C = the carbon absorption ratio.

**Table 4:** Carbon emissions from different land-use types in the PRD (10^4^ t C).

	Cultivated Land	Forest	Grassland	Other Agricultural Land	Residential, Mining and Manufacturing Land	Transport-ation Land	Water Bodies	Wetlands	Unused Land	Total
1990	8812.90	−17,301.68	−449.53	1616.57	7496.01	1992.61	−858.14	−621.41	−2.48	684.84
1995	8164.34	−17,442.12	−568.97	1497.60	9440.87	2509.60	−988.73	−715.97	−7.34	1889.29
2000	8005.05	−17,140.61	−430.87	1468.38	11,166.24	2968.24	−978.44	−708.53	−2.45	4347.01
2005	7267.29	−16,924.59	−402.25	1333.05	13,860.36	3684.40	−952.37	−689.65	−2.19	7174.05
2010	7083.24	−16,613.49	−417.65	1299.29	15,752.76	4187.44	−886.48	−641.94	−1.87	9761.31
2015	6966.62	−16,637.56	−439.20	1277.90	17,175.10	4565.53	−847.97	−614.04	−1.42	11,444.98

**Table 5:** The contribution value of each factor to land-use carbon emissions in the PRD (10^4^ t C).

Year	Land Use Change (∆*EI*)	Energy Structure Factor (∆*AI*)	Energy Efficiency Factor (∆*QI*)	Economic Development Factor (∆*GI*)	Population Factor (∆*P*)	Overall Effect(∆*C*)
1990–1995	785.08	−1378.65	117.98	1384.49	295.54	1204.45
1995–2000	1817.50	−1363.30	−100.23	1232.56	871.18	2457.72
2000–2005	1948.76	−3876.48	365.31	3612.75	776.71	2827.04
2005–2010	1612.48	−4838.04	−344.71	4759.05	1398.48	2587.26
2010–2015	642.49	−3246.59	−1020.82	4681.77	626.82	1683.67
1990–2015	6806.32	−14,703.05	−982.48	15,670.63	3968.73	10,760.14

## Data Availability

Not applicable.

## References

[1] Dirzo R., Young H.S., Galetti M., Ceballos G., Isaac N.J.B., Collen B. (2014). Defaunation in the Anthropocene. Science.

[2] United Nations, Department of Economic and Social Affairs, Population Division (2019). World Urbanization Prospects: The 2018 Revision, CA-ROM Edition.

[3] Weisz U., Pichler P.P., Jaccard I.S., Haas W., Matej S., Bachner F., Nowak P., Weisz H. (2020). Carbon emission trends and sustainability options in Austrian health care. Resour. Conserv. Recycl..

[4] IPCC 2014 IPCC Guidelines for National Greenhouse Gas Inventories. IPCC National Greenhouse Gas Inventories Programme..

[5] Meng Z.S., Wang H., Wang B.N. (2018). Empirical analysis of carbon emission accounting and influencing factors of energy consumption in China. Int. J. Environ. Res. Public Health.

[6] Yu X., Wu Z.Y., Zheng H.R., Li M.Q., Tan T.L. (2020). How urban agglomeration improve the emission efficiency? A spatial econometric analysis of the Yangtze River Delta urban agglomeration in China. J. Env. Manag..

[7] Wang S.J., Zeng J.Y., Huang Y.Y., Shi C.Y., Zhan P.Y. (2018). The effects of urbanization on CO_2_ emissions in the Pearl River Delta: A comprehensive assessment and panel data analysis. Appl. Energy.

[8] Zhao L.L., Huang W.J., Chen J.S., Dong Y.Y., Ren B.Y., Geng Y. (2020). Land use/cover changes in the oriental migratory locust area of China: Implications for ecological control and monitoring of locust area. Ag. Ecosyst. Environ..

[9] IPCC 2006 IPCC Guidelines for National Greenhouse Gas Inventories. IPCC National Greenhouse Gas Inventories Programme..

[10] Davis S.J., Caldeira K. (2010). Consumption-based accounting of CO_2_ emissions. Proc. Natl. Acad. Sci. USA.

[11] Wang C.J., Wang F., Zhang X.L., Yang Y., Su Y.X., Ye Y.Y., Zhang H.O. (2017). Examining the driving factors of energy related carbon emissions using the extended STIRPAT model based on IPAT identity in Xinjiang. Renew. Sustain. Energy Rev..

[12] Ma H.T., Sun W., Wang S.J., Kang L. (2019). Structural contribution and scenario simulation of highway passenger transit carbon emissions in the Beijing-Tianjin-Hebei metropolitan region, China. Resour. Conserv. Recy..

[13] Del Moretto D., Branca T.A., Colla V. (2018). Energy efficiency and reduction of CO_2_ emissions from campsites management in a protected area. J. Env. Manag..

[14] Foley J.A., DeFries R., Asner G.P., Barford C., Bonan G., Carpenter S.R., Chapin F.S., Coe M.T., Daily G.C., Gibbs H.K. (2005). Global consequences of land use. Science.

[15] Houghton R.A., Hackler J.L. (1999). Emissions of carbon from forestry and land-use change in tropical Asia. Glob. Chang. Biol..

[16] Lai L., Huang X.J., Yang H., Chuai X.W., Zhang M., Zhong T.Y., Chen Z.G., Chen Y., Wang X., Thompson J.R. (2016). Carbon emissions from land-use change and management in China between 1990 and 2010. Sci. Adv..

[17] Spawn S.A., Lark T.J., Gibbs H.K. (2019). Carbon emissions from cropland expansion in the United States. Envir. Res. Lett..

[18] Wu L.F., Liu S.F., Liu D.L., Fang Z.G., Xu H.Y. (2015). Modelling and forecasting CO_2_ emissions in the BRICS (Brazil, Russia, India, China, and South Africa) countries using a novel multi-variable grey model. Energy.

[19] Tongwane M., Mdlambuzi T., Moeletsi M., Tsubo M., Mliswa V., Grootboom L. (2016). Greenhouse gas emissions from different crop production and management practices in South Africa. Environ. Dev..

[20] Kirschbaum M.U.F., Saggar S., Tate K.R., Giltrap D.L., Ausseil A.G.E., Greenhalgh S., Whitehead D. (2012). Comprehensive evaluation of the climate-change implications of shifting land use between forest and grassland: New Zealand as a case study. Agric. Ecosyst. Environ..

[21] Chuai X.W., Huang X.J., Wang W.J., Zhao R.Q., Zhang M., Wu C.Y. (2015). Land use, total carbon emission’s change and low carbon land management in Coastal Jiangsu, China. J. Clean. Prod..

[22] Xia C.Y., Li Y., Xu T.B., Ye Y.M., Shi Z., Peng Y., Liu J.M. (2018). Quantifying the spatial patterns of urban carbon metabolism: A case study of Hangzhou, China. Ecol. Indic..

[23] Shao S., Chen Y., Li K., Yang L.L. (2019). Market segmentation and urban CO_2_ emissions in China: Evidence from the Yangtze River Delta region. J. Env. Manag..

[24] Zhang R.S., Matsushima K., Kobayashi K. (2018). Can land use planning help mitigate transport-related carbon emissions? A case of Changzhou. Land Use Policy.

[25] Baumann M., Gasparri I., Piquer-Rodrguez M., Pizarro G.G., Griffiths P., Hostert P., Kuemmerle T. (2017). Carbon emissions from agricultural expansion and intensification in the Chaco. Glob. Chang. Biol..

[26] Li B.B., Fang X.Q., Ye Y., Zhang X.Z. (2014). Carbon emissions induced by cropland expansion in Northeast China during the past 300 years. Sci. China Ser. D Earth Sci..

[27] Hudiburg T.W., Luyssaert S., Thornton P.E., Law B.E. (2013). Interactive effects of environmental change and management strategies on regional forest carbon emissions. Env. Sci. Technol..

[28] Liu L.N., Qu J.S., Maraseni T.N., Niu Y.B., Zeng J.J., Zhang L.H., Xu L. (2020). Household CO_2_ emissions: Current status and future perspectives. Int. J. Environ. Res. Public Health.

[29] Chen C., He X.Y., Liu Z.S., Sun W.W., Dong H., Chu Y.L. (2020). Analysis of regional economic development based on land use and land cover change information derived from Landsat imagery. Sci. Rep..

[30] Paul S., Saxena K.G., Nagendra H., Lele N. (2021). Tracing land use and land cover change in peri-urban Delhi, India, over 1973–2017 period. Environ. Monit. Assess..

[31] Enriquez-de-Salamanca A., Martin-Aranda R.M., Diaz-Sierra R. (2017). Potential of land use activities to offset road traffic greenhouse gas emissions in Central Spain. Sci. Total Environ..

[32] Lin B.Q., Raza M.Y. (2019). Analysis of energy related CO_2_ emissions in Pakistan. J. Clean. Prod..

[33] Ma X.J., Wang C.X., Dong B.Y., Gu G.C., Chen R.M., Li Y.F., Zou H.F., Zhang W.F., Li Q.N. (2019). Carbon emissions from energy consumption in China: Its measurement and driving factors. Sci. Total Environ..

[34] Ye L.L., Wu X.D., Huang D.D. (2018). Industrial energy-related CO_2_ emissions and their driving factors in the Yangtze River Economic Zone (China): An extended LMDI analysis from 2008 to 2016. Int. J. Environ. Res. Public Health.

[35] Roman R., Cansino J.M., Rodas J.A. (2018). Analysis of the main drivers of CO_2_ emissions changes in Colombia (1990-2012) and its political implications. Renew. Energy.

[36] Gonzalez P.F., Landajo M., Presno M.J. (2014). Tracking European Union CO_2_ emissions through LMDI (logarithmic-mean Divisia index) decomposition. The activity revaluation approach. Energy.

[37] De Oliveira-De Jesus P.M. (2019). Effect of generation capacity factors on carbon emission intensity of electricity of Latin America & the Caribbean, a temporal IDA-LMDI analysis. Renew. Sustain. Energy Rev..

[38] Yue W., Cai Y., Yang Z., Rong Q., Dang Z. (2018). Structural optimization for industrial sectors to achieve the targets of energy intensity mitigation in the urban cluster of the Pearl River Delta. Ecol. Indic..

[39] Adnan M.S.G., Abdullah A.Y.M., Dewan A., Hall J.W. (2020). The effects of changing land use and flood hazard on poverty in coastal Bangladesh. Land Use Policy.

[40] Liu J.Y., Kuang W.H., Zhang Z.X., Xu X.L., Qin Y.W., Ning J., Zhou W.C., Zhang S.W., Li R.D., Yan C.Z. (2014). Spatiotemporal characteristics, patterns, and causes of land-use changes in China since the late 1980s. J. Geogr. Sci..

[41] Searchinger T.D., Wirsenius S., Beringer T., Dumas P. (2018). Assessing the efficiency of changes in land use for mitigating climate change. Nature.

[42] West T.O., Marland G. (2002). A synthesis of carbon sequestration, carbon emissions and net carbon flux in agriculture: Comparing tillage practices in the United States. Agric. Ecosyst. Environ..

[43] Fang J.Y., Guo Z.D., Piao S.L., Chen A.P. (2007). Terrestrial vegetation carbon sink in China from 1981-2000. Sci. China Ser. D Earth Sci..

[44] Duan X.N., Wang X.K., Lu F. (2008). Carbon sequestration and its potential by wetland ecosystem in China. Acta Ecol. Sin..

[45] Ang B.W. (2005). The LMDI approach to decomposition analysis: A practical guide. Energy Policy.

[46] Wang W.W., Liu X., Zhang M., Song X.F. (2014). Using a new generalized LMDI (logarithmic mean Divisia index) method to analyze China’s energy consumption. Energy.

[47] Chunbo M., David I. (2008). China’s changing energy intensity trend: A decomposition analusis. Energy Econ..

[48] Popp A., Humpenoder F., Weindl I., Bodirsky B.L., Bonsch M., Lotze-Campen H., Muller C., Biewald A., Rolinski S., Stevanovic M. (2014). Land-use protection for climate change mitigation. Nat. Clim. Chang..

[49] Xu Q., Dong Y.X., Yang R. (2018). Urbanization impact on carbon emissions in the Pearl River Delta region: Kuznets curve relationships. J. Clean. Prod..

[50] Chen L., Xu L.Y., Yang Z.F. (2017). Accounting carbon emission changes under regional industrial transfer in an urban agglomeration in China’s Pearl River Delta. J. Clean. Prod..

[51] Foster G. (2020). Circular economy strategies for adaptive reuse of cultural heritage buildings to reduce environmental impacts. Resour. Conserv. Recy..

[52] Yang W.J., Zhao R.Q., Chuai X.W., Xiao L.G., Cao L.H., Zhang Z.P., Yang Q.L., Yao L.G. (2019). China’s pathway to a low carbon economy. Carbon Balance Manag..

[53] Moisen G.G., McConville K.S., Schroeder T.A., Healey S.P., Finco M.V., Frescino T.S. (2020). Estimating land use and land cover change in North Central Georgia: Can remote sensing observations augment traditional forest inventory data?. Forests.

